# Prediction of resilience based on parenting and coping strategies in patients with psychosomatic disorders

**DOI:** 10.1186/s40359-024-01784-9

**Published:** 2024-05-19

**Authors:** Sogand Sobhani, Hamidreza Jamilian, Iman Paknejad

**Affiliations:** 1 Master of Clinical Psychology, Faculty of Medical Sciences, Islamic Azad University, Iran Arak,; 2 Department of Psychiatry, Arak University of Medical Sciences, Arak, Iran

**Keywords:** Resilience, Parenting, Coping strategies, Psychosomatic disorders

## Abstract

The present research was conducted aiming at predicting the resilience based on parenting and coping strategies in patients with psychosomatic disorders. The statistical population of the present research consisted of all patients suffering from psychosomatic disorders who had visited medical clinics related to Medical Sciences (University) of Arak County in 2019-20. The statistical sample includes 347 women, aged 18 to 55 years-old, who were selected by available sampling. Data collection tool included Young parenting, Moss and Billings coping strategies questionnaire and Connor and Davidson resilience questionnaire. Pearson’s correlation coefficient and multiple regression analysis were used to analyze the data. The findings showed that parenting (dependency, preoccupied/untransformed self) have a negative and significant relationship with resilience. Coping strategies focused on emotion, coping focused on physical restraint or physicalization of problems have a negative and significant relationship with resilience and have a positive and significant relationship with coping strategies focused on problem-solving and coping focused on cognitive evaluation. According to the findings, it can be concluded that it is possible to pave the way for increasing resilience and preventing the development of psychosomatic disorders by creating suitable conditions during the childhood, improving parent-child relationships, and by strengthening coping strategies focused on problem-solving and coping focused on cognitive evaluation.

## Introduction

Some physical diseases can be worsened by psychological factors such as stress and anxiety. For example, psoriasis, eczema, stomach ulcer, high blood pressure, and heart disease. It seems that the actual physical problems of the disease (the extent of the impacts of psoriasis, blood pressure level, etc.) can be influenced by psychological factors. It is very difficult to prove this hypothesis. However, a large number of people with this disorder and other physical diseases say that their current mental state can affect their physical state at any given time [1].

Until the early 80s, many psychological researchers believed that stressful events play an effective role in the emergence of psychosomatic disorders. Holmes & Rahe [2] found that life events are related to the onset of the disease and there are moderating factors between stressful events and psychological disorders that cause these events to have different impacts on people. One of these characteristics is resilience. Some viewpoints consider the resilience as a response to a specific event and some others consider it as a sustainable coping strategy [3].

The impacts of psoriasis may not bother some people very much while trying to cover up these impacts in another person leads to increased feelings of depression and illness. The physical impacts can be caused by mental illness. Some psychological diseases may cause anorexia nervosa, or excessive self-care, which causes physical problems. However, the term psychosomatic disorder mainly means a physical disease that is thought to be caused or worsened by psychological factors. People, who experience stressful events such as trauma, abuse, frequent illness, fear, depression, anger, sense of guilt, insecurity, and other difficult conditions, are also susceptible to this condition [4].

Resilience is defined as the effective negotiation process, adaptation to, or managing major sources of stress or trauma. Current models of mental resilience show that factors can be classified as internal (genetic) and external (environmental). Internal factors are created from within a person and include biological and psychological factors. The external factors are the ones that are reflected in the nature and quality of relationships established inside and outside the family group [5].

Coping strategies include a person’s cognitive, emotional, and behavioral efforts to control external and internal behaviors that threaten or challenge a person and have a two-way relationship with mental health. According to Lazarus [6], coping is a process in which people contain the tensions caused by stressors and control the negative emotions created by these factors. Using uncompromised strategies in the long term leads to loss of confidence in the ability to face challenges, anxiety, physical diseases, and depression. Every person experiences various medical diseases due to the mental stress. Physical diseases caused by psychological factors can be treated through medicine or surgery, but the complete treatment of this disease can only be achieved if the cause of creation of psychological pressures is determined. Therefore, treatment methods to reduce these psychological factors such as stress, anxiety, depression, etc. are necessary to improve the physical illness. Psychological factors can partially change a medical condition. Studies have shown that psychological stress can affect the growth and spread of a tumor and thereby worsen the cancer condition. Some medical conditions are caused by mental stress, such as blood pressure, which is directly related to psychological factors such as negative emotional state, frequent stress, and social factors such as economic status and life events [4].

The parents’ beliefs, attitudes, activities, and actions manifest in the form of family patterns or parenting styles of parents, and the fact that which of the different parenting styles are adopted by parents in the family is in itself influenced by various factors that originate from culture and society [7]. Sound parenting skills are key predictors of positive outcomes for children in the early and middle years of life [8].

Parents’ performance has a significant impact on the formation of children’s thoughts, behavior, and emotions [9]. Studies have indicated that the quality of parenting has a great impact on the natural development of a child. Also, some factors such as family conflicts and marriage failure, not having a warm relationship with parents, insecure attachment, strict regulations, insufficient supervision, and psychiatric diseases in parents, increase the risk of emotional and behavioral problems development in children [10].

Family conditions including parental absence, parents’ behavior towards children, and problems in relationship are also the main source of psychosomatic disorders [4].

Parenting refers to special behaviors and methods that affect the child’s development separately or in interaction with each other. In fact, the basis of the parenting method is the parents’ efforts to control and socialize their children [11].

Among the parenting styles, three important and major styles can be mentioned, which include authoritarian, despotic, and permissive styles. In authoritarian parenting, the rules and norms are clearly explained to the children and the parents encourage the children to follow the rules and norms with reasoning. Parents deal with children’s negligence with lenient and involve them in family decisions. In despotic parenting, there is a lot of control and supervision of children and there is no accountability for them in contrast. Strict and inflexible rules and norms are imposed on children and they have to obey their parents unconditionally [12].

There is little control and supervision over children and a lot of accountability to them in permissive parenting. Parents apply few behavioral rules and norms to control and supervise their children, and since children face few rules and norms, they find the possibility to do what they want or desire. In this style, due to the low control and supervision of the parents, the children have a lot of freedom of action, and this freedom of action leads to a great accountability of the parents to the children, and the parents consider themselves responsible for responding to the children’s requests [13, 14].

Studies have concluded that the development of coronary heart disease is related to several risk factors, of which depression, anxiety, and stress are the main ones. Respiratory problems, apart from various etiological influences, emotional stress is the main trigger factor associated with induction of bronchial asthma. Gastrointestinal problems or stomach ulcer formation is related to stressful events in a person’s life. Since this disorder is related to mind and body, its treatment also includes medical actions from two psychological and medical wards. Before providing the appropriate treatment, the stressful factors of the person suffering from psychosomatic disorder are checked first [4].

Children’s resilience in environmental and family conditions is related to their parents’ upbringing style, and the way parents raise children can be an important factor in increasing their resilience and resistance to problems. Parenting styles are decisive and influential factors that play an important role in children’s psychopathology and development [15].

Sikand [16] conducted a research in the field of parenting styles, cognitive style, and resilience of women with dissociative disorder. They concluded that there is a significant relationship between emotional warmth and systematic-cognitive style and between systematic-cognitive style and high resilience.

Zhong et al. [5] investigated parenting styles, resilience, and mental health of the elderly in a research in China. The findings showed that the elderly, who had a positive and authoritative parenting style, showed a high level of mental resilience and a low level of depression and anxiety. The elderly, who grew up in families with authoritarian parenting style, had high levels of anxiety and depression and low mental resilience.

Kritzas & Grobler [15] conducted a research on the relationship between parental understanding styles and resilience in adolescence periods. The findings indicated that there is a positive relationship between fathers’ authoritarian styles and strategies coping with focused feelings on adolescent resilience.

McCain et al. [17] conducted a research titled the study of the relationship between resilience, job burnout, and coping strategies in physicians. The average resilience was 68.9% higher than the population norms. 100 (37%) physicians had high job burnout, 194 (72%) of them had high secondary trauma stress. They had high secondary psychological pressure and 64 people (24%) had low compassion satisfaction. Burnout was associated with low resilience, low compassionate satisfaction, secondary traumatic stress, and frequent use of maladaptive coping mechanisms, including self-blame, disengagement, and substance use. Non-clinical issues in the workplace have been the main perceived cause of low resilience in physicians. Pan [18] conducted a research titled resilience and coping strategies affecting the quality of life in brain tumor patients. The results showed that there is a negative and significant correlation between resilience and uncertainty about the future and motor (motion) dysfunction. In addition, there is a significant negative correlation between emotion-focused coping and uncertainty about the future, as well as between problem-focused coping and motor dysfunction. Prediction of uncertainty of future quality of life of this study emphasizes the potential importance of resilience and coping strategy in patients’ quality of life, which is related to brain tumor treatment. Kritzas & Grobler [15] found in a research that there is a positive relationship between coping strategies and focused feelings in adolescent resilience.

By explaining the relationship between coping strategies and resilience, this study can enhance the levels of resilience in patients with psychosomatic disorders and improve the treatment methods of patients with psychosomatic disorders. It is also able to compile (develop) a protocol for patients, who go to hospitals and medical centers, to modify the treatment methods of psychosomatic patients.

Considering the importance of resilience in dealing with the countless stresses of life, the current research was conducted with the aim of predicting the resilience based on parenting and coping strategies in patients with psychosomatic disorders. Therefore, the main question of this research is whether it is possible to predict resilience based on parenting and coping strategies in patients with psychosomatic disorders?

## Methods

According to the subject and purpose of the research descriptive, the methodology of the research is correlational and quantitative in terms of the type of data, and the instrument used is a questionnaire. The target population in this research is all patients with psychosomatic disorders who visited the medical clinics affiliated to Arak University of Medical Sciences in 2019–2020. 347 women, aged 18–55 years, suffering from psychosomatic disorders were selected and investigated using available sampling method in this research.

Most of these women were young and had university educations who had referred to the clinics covered by the University of Medical Sciences in Arak city for the treatment of psychosomatic problems.

## Ethics approval and consent to participate

### Ethical approval

for this study was obtained from the Arak University of Medical Sciences Ethics committee (IR.IAU.ARAK.REC.1399.030; arakmu.ac.ir).

## Research tool

### Connor and davidson resilience scale (CD-RISC)

Connor & Davidson Resilience Scale [19] contains 25 items that are scored on a Likert scale between zero (completely false) and four (always true). The minimum resilience score of the subject in this scale is zero and the maximum score is 100. Although this scale measures different aspects of resilience, it has a total score.

In a research that was conducted on 641 Spanish university students between the ages of 14 and 30, the Cronbach correlation coefficient was 0.85 and the test-retest correlation coefficient was 0.71 [20]. The results indicated that the Connor and Davidson Resilience Scale (CD-RISC) can be used as a valid instrument to measure resilience. The validity and reliability of this questionnaire has been confirmed in Iran [21].

### Young parenting questionnaire

The questions of this questionnaire are a reflection of the childhood environment. This self-report questionnaire consists of 72 statements that people may use to describe their parents. Each statement reflects parenting behaviors that are associated with one of 17 negative core beliefs (primary maladaptive schemas). Each of the 72 items are rated on two six-point Likert scales (1: completely false, 2: very false, 3: somewhat false, 4: almost true, 5: very true, 6: absolutely true), which reflects how well each statement describes the participants’ parents. Except for the emotional deprivation scale, which is scored inversely, 1: “completely false” and 6: “completely true”, higher scores indicate that parents have acted in ways that are more likely to create relevant core beliefs [22]. Cronbach’s alpha reliability values ranged from 0.67 to 0.92. Acceptable test-retest reliability and correlations ranged from 0.53 to 0.85 [23]. In an Iranian research, the original form of Young parenting questionnaire was translated and then implemented on 60 Iranian students. Then, the validity and reliability of the mother form and the father form was obtained through the split–half method [24].

### Billings and moss coping strategies questionnaire

Billings and Moss Coping Strategies Questionnaire contains 19-item yes/no questionnaire according to how they were involved with that event. Coping responses were divided into active cognitive, social, and active behavioral studies according to apparent validity. In this case, internal consistency validity for the three subscales are reported from 0.44 to 0.80. Although no significant correlation was found between severity and coping life event, coping comments significantly increased the predictive power of stress levels. The two (Billings and Moss) followed up their work in 1984 by investigating the coping behaviors in a group of depressed patients. They increased the number of coping responses to 32 items and used a 4-point Likert scale instead of yes/no. According to this choice, 0 to 3 points are assigned to the person’s response. 5 types of coping strategies were identified in this new questionnaire: 5 items related to coping focused on cognitive evaluation, 3 items related to coping focused on problem-solving, 11 items related to coping focused on emotion, 4 items related to coping based on gaining social support, and 9 items related to coping focused on physical restraint or physicalization of problems. In testing for reliability, the CSQ-D as a whole had a Cronbach’s alpha of 0.94 and an intraclass correlation coefficient of 0.89 (95% CI 0.86–0.98). The total CSQ-D score was correlated to the FESV-BW scales with scores of *r* = 0.32–0.55 and with the SF-36 Mental Component Summary with scores of *r* = 0.32–0.503 [25]. The validity and reliability of Persian version of Moss-Billings Stress Coping Strategies Scale was assessed in women [26].

The data gathered have been analyzed using SPSS V. 26 software with regression and Pearson correlation methods. In the regression analysis, resilience as a dependent variable and parenting styles and coping styles were entered into the model as independent variables. We have used the central limit theorem in order to check the normality of the data related to each variable due to the high sample size. The normality was checked by Shapiro-Wilk test.

### Hypothesis of this research

We hypothesize that the resilience and coping strategies of patients with psychosomatic disorders is predicted by parenting.

## Findings

### Description of demographic indicators

The frequency distribution of age and level of education of the participants are reported in Table 1:

**Table 1:** Frequency distribution of demographic variables

Variable	Group	Frequency	percent
Age	25 years	43	12
	26–35 years	209	60
	36–45 years	89	25
	46–55 years	6	3
Education	High school	1	1
	diploma	27	8
	Bachelor’s degree	140	40
	Master’s degree	133	38
	PhD	46	13

The results of Table 1 show that most of the participants have university education at the bachelor’s, master’s, and doctoral levels, and most of them are in the age group of 26 to 35 years.

### Data normality test

We have used the central limit theorem in order to check the normality of the data related to each variable due to the high sample size. We did this in such a way that the sample size was divided into 10-individual groups, the average was taken, and their normality was checked by Shapiro-Wilk test, the results of which are presented in Table 2:

**Table 2:** Shapiro-wilk-wilk test results

Variable	Statistics	df	Sig.
Resilience	0.992	34	0.070
Parenting	0.995	34	0.334
Coping Strategies	0.982	34	0.840

The multivariate regression model has been used simultaneously to test the hypotheses after checking the presuppositions and confirming them to use the regression model.

### Testing of hypothesis

First, the correlation matrix between research variables is presented in Table 3 in this section.

**Table 3:** Matrix of correlation coefficients between resilience variables and coping strategies

Variable	Resilience	Cognitive assessment	Problem- Solving	Excitement	Winning social support	Physicalization of problems
Resilience	1					
Coping Strategies						
Cognitive Assessment	0.587 **	1				
Problem-Solving	0.694 **	0.384 **	1			
Excitement	0.338 **	0.146 **	0.332 **	1		
Winning social support	0.193**	0.151 **	0/024-	0.307 **	1	
Physicalization of problems	0.279 **	0.0370	0.095	0.447 **	0.304 **	1

*The correlation is significant at the 0.05 level

** The correlation is significant at the 0.01 level

The continuation of the correlation matrix between research variables is presented in Table 4.

**Table 4:** Matrix of correlation coefficients between resilience variables and parenting styles

Variable	1	2	3	4	5	6	7	8	9
1- Resilience	1								
Young parenting									
2-Emotional deprivation	0.038	1							
3-Abandonment	*0.108	0.034	1						
4- Distrust	0.119-*	0.034	0.602**	1					
5- Vulnerability to disease	0.104	0.146**	0.009	0.049	1				
6-Incompetence	0.230**	0/030-	0.465**	0.446**	0.121*	1			
7- defects	0.212-**	0/041	0.620**	0.572**	0/012-	0.733**	1		
8- Failure	0.193-**	042/. -	0.582**	0.635**	0/017	0.647**	0.788**	1	
9- Obedience	0.168**	0.003	0.566**	0.523**	0.023	0.768**	0.797**	0.755**	1
Variable	10	11	12	13	14	15	16	17	18
10-Sacrifice	1								
11- Stubborn standards	0.545**	1							
12- Grand secretary	0.523**	0.775**	1						
13- You have yourself	0.437**	0.475**	0.595**	1					
14-Caught	0.380**	0.555**	0.507**	0.452**	1				
15-negativity	0.480**	0.726**	0.676**	0.578**	0.529**	1			
16-Emotional inhibition	0.455**	0.680**	0.603**	0.532**	0.585**	0.633**	1		
17- Punishment	0.414**	0.715**	0.703**	0.505**	0.436**	0.760**	0.564**	1	
18- Acceptance	0.457**	0.725**	0.643**	0.445**	0.541**	0.621**	0.630**	0.619**	1

*The correlation is significant at the 0.05 level

** The correlation is significant at the 0.01 level

#### Hypothesis 1

Resilience of patients with psychosomatic disorders based on parenting (emotional deprivation, abandonment, mistrust, vulnerability, dependence, defect, failure, obedience, selflessness, stubborn criteria, entitlement, restraint, trapped, negativity, emotional inhibition, punishment, and seeking acceptance) is predicted.

A Summary of the Regression Model:

**Table 5:** Regression summary

Model	*R*	*R* Square	Adjusted *R* Square	Std. Error of the Estimate
1	0.270	0.073	0.059	0.611

According to the results of Table 5, the adjusted R square value shows that the used model accounted for 0.059 change in resilience scores.

**Table 6:** Significance test of regression model

Model	Sum of Squares	df	Mean Square	F	Sig.
Regression	9.969	5	1.994	334.5	0.001
Residual	342.126	338	0.374		
Total	311.136	343			

In Table 6, the results of the analysis of variance show that the regression model has a good fit. The obtained F value is equal to 5.334, which is significant at the 0.001 level.

**Table 7:** Table of coefficients related to regression variables

Model		Unstandardized Coefficients		Standardized Coefficients	t	Sig.
		B	Std. Error	***Beta***		
***1***	(Constant***)***	2.643	0.092		***28.758***	***0.001***
	Shame and imperfection	-0.030	0.024	-0.129	***-1.261***	***0.208***
	Failure	-0.021	0.026	-0.072	***-0.801***	***0.424***
	Dependency	-0.036	0.019	-0.172	***-1.964***	***0.050***
	Obedience	0.036	0.023	0.164	***1.589***	***0.113***
	Busy	-0.033	0.016	-0.115	***-2.007***	***0.046***

As shown in Table 7, two components of dependency (*P* < 0.050) and caught (*P* < 0.046) with a negative regression coefficient are significantly influential in resilience prediction. Therefore, he formulated the prediction model as follows:

Y = 2. 0-643. 050* Dependency value-0. 046* value of busy.

#### Hypothesis 2

Resilience of patients with psychosomatic disorders based on coping strategies (coping focused on cognitive evaluation, coping focused on problem-solving, coping focused on emotion, coping focused on gaining social support, coping focused on physical restraint or physicalization of problems) is predicted.

Summary of the regression model:

**Table 8:** Regression summary

Model	*R*	*R* Square	Adjusted *R* Square	Std. Error of the Estimate	Durbin-Watson
1	0.756	0.572	0.565	0.415	1.785

According to the results of Table 8, the adjusted R square value shows that the used model accounted for 0.565 change in resilience scores.

**Table 9:** Significance test of the regression model

Model	Sum of Squares	df	Mean Square	F	Sig.
Regression	77.943	5	15.589	90.272	0.001
Residual	58.368	338	0.173		
Total	136.311	343			

In Table 9, the results of the analysis of variance show that the regression model has a good fit. The obtained F value is 90.272, which is significant at the 0.001 level.

**Table 10:** Table of coefficients related to regression variables

Model		Unstandardized Coefficients		Standardized Coefficients	t	Sig.
		B	Std. Error	***Beta***		
***1***	(Constant***)***	***1.084***	***0.138***		***7.843***	***0.001***
	Cognitive assessment	***0.426***	***0.064***	***0.292***	***6.658***	***0.001***
	Problem-Solving	***0.440***	***0.044***	***0.462***	***10.104***	***0.001***
	Excitement	***-0.183***	***0.072***	***-0.105***	***-2.519***	***0.012***
	Attracting social support	***0.049***	***0.043***	***0.042***	***1.152***	***0.250***
	Physicalizing problems	***-0.166***	***0.053***	***-0.128***	***-3.133***	***0.002***

As shown in Table 10, the two components of coping focused on cognitive evaluation (*P* < 0.001) and coping focused on problem-solving (*P* < 0.001) have a positive and significant relationship and coping focused on emotion (*P* < 0 0.05) and coping focused on physical restraint or physicalization of problems (*P* < 0.002) have a negative relationship and a significant power in resilience prediction.

## Discussion and conclusion

Considering the importance of resilience as a subject that deals with mental health with a preventive approach in psychology, this research was conducted aiming at predicting the resilience of patients with psychosomatic disorders based on parenting and coping strategies. The results of this study showed that resilience has a significant correlation with components of coping focused on cognitive evaluation, coping focused on problem-solving, coping focused on emotion, gaining social support, coping focused on physical restraint or physicalization of problems. In addition, the components of the coping strategies that predicts the resilience include: Coping focused on problem-solving, coping focused on cognitive evaluation, and coping focused on physical restraint, and coping focused on emotion. Therefore, the first hypothesis was confirmed and it was proved that resilience can be predicted based on coping styles.

The findings of this study are in line with the results of similar research such as Kritzas & Grobler [15] and Pan [18]. These researches considered coping strategies as predictors of resilience, too.

In explaining this finding, if we look at coping methods based on schema-therapy, then we can see that coping responses and behavior originate from schemas. Coping strategies and parenting can appear cognitively, emotionally, or behaviorally, and since the underlying schemas of these coping styles are often formed during the childhood and adolescence and have a strong emotional element, under stressful conditions and when the people’s schemas get active, the related emotions get also activated, and most of these emotions and physical feelings are biologically stored in the amygdala system, and these emotions are unconsciously and automatically activated in these conditions and lead to the emergence of relevant confrontation or behavior before cognitive processing in the higher areas of the cerebral cortex. The fact that the emotional and cognitive aspects of stressful and upsetting experiences are stored in different areas of the brain may explain why only cognitive methods and behavioral skill training (such as problem-solving method) can cause deep changes [27].

Also, the results of this study showed that resilience is correlated with the components of shame and failure, untransformed/trapped self, obedience, dependence, and failure, and parenting styles predicting the resilience are as follows, respectively: Trapped/untransformed self, and dependence. Therefore, this hypothesis was also confirmed and it was proved that resilience can be predicted based on parenting styles. The findings of this study are consistent with the results of similar studies, including Sikand [16], Zhong et al. [5], Kritzas & Grobler [15]. This research also considered parenting styles as predictors of resilience.

The aspect of abandonment, emotional deprivation, shame/deficiency, and emotional inhibition can predict emotional disorders in children. Parenting practices in the five areas, except for the other-directedness area, are good predictors for maladaptive schemas in the two areas of cut-off-rejection and self-management-impaired performance. Among the three parenting styles, there is a positive and significant relationship between authoritarian parenting style and students’ learning problems. Also, there was a negative and significant relationship between parents’ psychological resilience and students’ learning problems. In addition, the despotic parenting style and resilience were also significant predictors of students’ learning problems. There is a significant relationship between emotional warmth and systematic-cognitive style and between systematic-cognitive style and high resilience [16]. The elderly, who had a positive and authoritative parenting style, showed a high level of mental resilience and a low level of depression and anxiety. The elderly, who grew up in families with despotic parenting style, had high levels of anxiety and depression and low mental resilience [5]. There is a positive relationship between fathers’ authoritarian styles and emotion-focused coping strategies in white adolescents’ resilience. In contrast, other researchers found that authoritarian and harsh parenting styles are closely related to mental disorder [15].

If someone has a certain temperament and at the same time, his/her needs are not met at a certain time, then he/she will experience one or more of the schemas or personality traps. For example, a child, who grew up in an insecure family and did not see enough affection from the people around him/her, will suffer from a lack of affection in adulthood and may be severely shaken in emotional relationships and look for a partner from time to time or even vice versa take a distance from others and all these are the consequences of lack of affection. According to what experiences a person has had and how their needs have been met, and what is a person’s temperament, five areas have been identified, each of which includes some schemas: 1- Cut-off and rejection 2- Self-management and impaired performance 3- impaired limitations 4- other-directedness 5- excessive vigilance and inhibition [27]. The person with dependency schema and trapped/untransformed self includes the second area of self-management and impaired function. A person’s expectations of himself/herself and the environment interfere with his/her sensible abilities to separate, survive, and function independently or to accomplishing the tasks successfully. The families of these individuals usually have the following characteristics: they reduce the child’s self-confidence, they are busy, they protect the child too much and they have not been able to encourage the child to do things outside the family [27]. Investigations show that physical disorders related to mental stress are due to hyperactivity of impulses sent from the brain to other parts of the body, which can cause the release of adrenaline in the blood and lead to a level of anxiety. Changes in glucose metabolism, amino acid levels in serum, etc. can cause psychosomatic disorders. Also, people, who experience stressful events such as trauma, abuse, frequent disease, fear, depression, anger, guilt, insecurity and other difficult situations are also prone to this condition. Absence of parents, parents’ behavior towards children and relationship problems are also the main origin of psychosomatic disorders [4]. So, it can be concluded that one of the influential factors in creating psychosomatic disorders is parenting (busy, untransformed self, dependency) and an effective step can be taken to increase the resilience in order to prevent psychosomatic disorders by modifying parenting methods and improving parent-child relationships. Furthermore, it is possible to strengthen coping strategies focused on problem-solving and coping focused on cognitive evaluation to prevent the occurrence of psychosomatic disorders and increase resilience.

The limitations of conducting this research are the inherent limitations of using the questionnaire tool and selecting the research sample that is available sampling because this reduces the results generalization power. Another limitation was that only women with high levels of education were studied - including the group of 46 women with PhD. Based on the obtained results, it seems that the way can be paved to increase the efficiency of people in effectively dealing with stressful situations by providing suitable conditions during childhood, improving parent-child relationships, and providing training courses on coping strategies. Therefore, it is suggested to think of measures to teach (train) efficient parenting methods and to improve coping strategies in order to enhance the resilience in patients with psychosomatic disorders, and to develop protocols for clients to hospitals and treatment centers to improve psychosomatic patients. It is suggested to conduct this research for men and women of different ages and other parts of the country or in a longitudinal way. It is recommended to examine the interrelationships of resilience with life satisfaction, treatment satisfaction and marital satisfaction in future research. Also, due to the fact that only women with high education have participated in this research, it is suggested that in future researches, the subject of this study should be investigated on men or women with low education.

## Data Availability

The data that support the findings of this study are available on request from the corresponding author, [initials]. The data are not publicly available due to [restrictions e.g. their containing information that could compromise the privacy of research participants].
